# Effects of Tocilizumab, an Anti-Interleukin-6 Receptor Antibody, on Serum Lipid and Adipokine Levels in Patients with Rheumatoid Arthritis

**DOI:** 10.3390/ijms20184633

**Published:** 2019-09-18

**Authors:** Elinoar Hoffman, Michal A. Rahat, Joy Feld, Muna Elias, Itzhak Rosner, Lisa Kaly, Idit Lavie, Tal Gazitt, Devy Zisman

**Affiliations:** 1 Rheumatology Unit, Carmel Medical Center, Haifa 3436212, Israel; elinoard@gmail.com (E.H.); joyfeld@gmail.com (J.F.); MUNAEL@clalit.org.il (M.E.); tgazitt@gmail.com (T.G.); 2 The Immunotherapy Laboratory, Carmel Medical Center, Haifa 3436212, Israel; mrahat@netvision.net.il; 3 The Ruth and Bruce Rappaport Faculty of Medicine, Technion, Haifa 3109601, Israel; itzhak.rosner@b-zion.org.il (I.R.); lavi_idit@clalit.org.il (I.L.); 4 Rheumatology Unit, Bnai Zion Medical Center, Haifa, 3339419, Israel; lisakaly@yahoo.fr; 5 Department of Community Medicine and Epidemiology, Carmel Medical Center, Haifa 3436212, Israel

**Keywords:** rheumatoid arthritis, tocilizumab, lipids, adipokines, adiponectin, resistin, leptin

## Abstract

Patients with rheumatoid arthritis (RA) are at increased risk of cardiovascular disease. Dyslipidemia is a known adverse effect of tocilizumab (TCZ), an anti-interleukin-6 receptor antibody used in RA treatment. We aimed to assess the effect of TCZ on lipid profile and adipokine levels in RA patients. Height, weight, disease activity scores, lipid profile and atherogenic indices (AI), leptin, adiponectin, resistin, interleukin-6, and high-sensitivity C-reactive protein (CRP) were measured before and four months after initiation of TCZ in 40 RA patients and 40 healthy controls. Following TCZ treatment, total cholesterol, high density lipoprotein (HDL), and triglycerides were significantly elevated, but no significant changes in weight, body mass index (BMI), low density lipoprotein (LDL), and AI were observed. Compared with controls, significantly higher adiponectin levels were measured in the RA group at baseline. Following TCZ treatment, resistin levels and the leptin-to-adiponectin ratio increased, adiponectin levels decreased, and leptin levels remained unchanged. No correlation was found between the change in adipokine serum levels and changes in the disease activity indices, nor the lipid profile. In conclusion, the changes observed suggest a protective role for TCZ on the metabolic and cardiovascular burden associated with RA, but does not provide a mechanistic explanation for this phenomenon.

## 1. Introduction

Rheumatoid arthritis (RA), a chronic inflammatory disease with typical bone erosion and damage, is also associated with an increased incidence of metabolic syndrome and cardiovascular disease (CVD). Although CVD risk factors such as obesity, smoking, hypertension, and diabetes are also increased in RA patients, systemic inflammation caused by RA itself seems to contribute to CVD risk in these patients. Chronic low-grade inflammation—often assessed by C-reactive protein (CRP), an acute phase reaction protein—is associated with accelerated atherosclerosis, increased CVD risk, and increased mortality [1]. Hence, it is suggested that successful treatment of RA patients so as to reduce the inflammatory burden may decrease CVD risk [2,3,4]. This has already been shown for the administration of tumor necrosis factor-alpha (TNF-α) inhibitors [5].

Adipokines, cytokines secreted mostly by white adipose tissue, are involved in inflammation, endothelial dysfunction, and atherosclerosis, placing them as possible molecular links between RA, the metabolic syndrome, and CVD risk [2,6,7,8]. However, their putative role as key inflammatory mediators contributing to CVD risk is still controversial, as their serum levels vary. For instance, the pro-inflammatory adipokine leptin has been shown to variably exhibit either elevated or similar serum levels in RA patients relative to healthy controls, while resistin, another pro-inflammatory adipokine, has been shown to have either elevated, similar, or reduced levels relative to healthy controls. Surprisingly, the serum and synovial fluid level of the anti-inflammatory adipokine adiponectin was found to be consistently elevated in RA patients [3,4,6,7,9,10,11,12,13]. Notably, the levels of these three major adipokines—leptin, resistin, and adiponectin—were shown to correlate with insulin resistance. The leptin-to-adiponectin ratio (LAR) is used to assess insulin resistance among type-2 diabetes mellitus patients and healthy individuals [14,15,16,17,18], while resistin levels were found to correlate with insulin resistance in septic patients [6].

Tocilizumab (TCZ), a human monoclonal antibody that targets the interleukin-6 (IL-6) receptor, is a novel biologic disease-modifying anti-rheumatic drug (bDMARD) effective in treating RA in humans [19]. TCZ has been shown to worsen dyslipidemia, a significant adverse metabolic effect that was attributed to the blockage of IL-6 and its ability to induce the expression of apolipoproteins in the liver [20]. However, despite that dyslipidemia is known to be associated with TCZ treatment, this biologic agent was not found to increase CVD risk in RA patients [21]. We hypothesized that TCZ might affect adipokine levels, thus exerting a cardioprotective role in RA patients.

Our aim in the present study was to evaluate the effects of TCZ treatment on serum lipid and adipokine levels in RA patients compared with healthy controls, and to evaluate whether any changes in adipokine profile mediated by TCZ are cardioprotective.

## 2. Results

### 2.1. Patient Characteristics

The average age of the 40 patients in the RA group was 57.5 years, and 82.5% were women, with a mean disease duration of 7.7 ± 5.6 years. All participants completed the four month follow-up. Their demographic and clinical characteristics are summarized by disease activity class (DAS28-CRP score) at baseline (Table 1).

There were no significant differences between the groups with respect to demographics, clinical parameters, comorbidities, and medical regimen.

The majority of the patients responded clinically to TCZ after four months of treatment (Table 2) with a reduction in disease activity as assessed by CDAI (25 patients, 62.5%) or DAS28-CRP (22 patients, 55%).

Following four months of treatment, 27.5% and 15% of the patients achieved low disease activity or remission by CDAI and DAS28-CRP criteria, respectively. There were no significant changes in concomitant medications including doses of corticosteroids, statins, or other disease modifying agents (DMARDs) during the four months of follow-up.

The effects of TCZ treatment on serum samples are summarized in Table 2. Following four months of treatment, CRP and hsCRP levels decreased significantly, while IL-6 levels increased significantly, demonstrating the predictable pharmacological effect of TCZ.

### 2.2. TCZ Effects on Metabolic Parameters

Upon recruitment, 25 RA patients were overweight (BMI > 25) and 11 were obese (BMI > 30). There were no significant changes in weight or BMI following four months of treatment. Total cholesterol, HDL, and TG levels increased significantly following treatment, with the increase in LDL not reaching statistical significance. Of note, three patients had incalculable LDL levels owing to hypertriglyceridemia. Atherogenic indices did not change significantly following treatment (Table 2).

### 2.3. TCZ Effects on Adipokine Levels

Before TCZ treatment, adiponectin levels adjusted to BMI and statin treatment were higher in RA patients compared with healthy controls (*p* < 0.0001), whereas LAR was decreased (*p* = 0.03) (Table 3). TCZ treatment normalized the adiponectin and resistin levels, which decreased to the levels observed in the healthy control group. Only leptin levels continued to increase after four months of treatment.

Following four months of TCZ treatment, significant changes in the levels of adiponectin, resistin, and LAR were noted after adjustment to BMI, statin treatment, and disease duration. Adiponectin levels decreased (*p* ≤ 0.0001), whereas resistin and LAR increased. The adipokine profile following four months of TCZ treatment trended to the levels measured in the control group, and no statistically significant differences were found between the patient group after treatment and controls in the three adipokine levels or the LAR measured in the study (Table 3).

The changes in the serum levels of the three adipokines prior to starting TCZ treatment and after four months of treatment did not correlate with the changes in the clinical and metabolic parameters that are associated with the risk of CVD (Table 4). The only significant correlation we found was a positive correlation between the changes in HDL values and levels of leptin.

There were no statistically significant differences in the adipokine levels when patients were stratified into responders and non-responders according to DAS28-CRP or CDAI score response after adjustment to BMI, statin treatment, and disease duration.

## 3. Discussion

In this study, we show that TCZ treatment improves disease activity and reduces the inflammatory burden in RA patients, as has been shown before. As expected, disease activity scores were significantly reduced with TCZ treatment, as were the values of hsCRP, a measure considered to reflect vascular inflammation and that serves as a predictor of cardiovascular events [1]. However, this improvement in disease activity was accompanied by increased dyslipidemia, as previously reported for TCZ. Therefore, we asked whether adipokines, which are the hypothesized link between inflammation and increased CVD risk, were responsible for this effect. Contrary to this hypothesis, we show that there is no correlation between changes in adipokine serum levels as a result of TCZ treatment and changes in the disease activity or lipid profile, indicating that the adipokines we examined do not directly regulate these parameters in our cohort.

Our RA study population was more overweight and obese (60% and 27.5%, respectively) than the general population in Israel (49% and 16%, respectively, in 2012) [22], and the four months of treatment with TCZ did not affect their BMI. The increased prevalence of obesity among our RA patients may be explained by the metabolic impact of the inflammatory state, which limits physical activity, as well as by prolonged corticosteroid treatment. However, the effects of TCZ on obesity are still controversial. Similar to our study, other studies found no significant change in weight or BMI in TCZ-treated non-diabetic RA patients over a period of three [23] or six months [24], or when patients were stratified to responders versus non-responders according to the DAS28-CRP criteria [25]. In contrast, Younis et al. demonstrated a significant rise in weight and BMI in TCZ-treated RA patients over a period of four months [26]. As TCZ blocks IL-6 signaling and leads to its elevated serum levels, as we demonstrated here, the metabolic effects of increased IL-6 should be taken into consideration. Elevated IL-6 levels in cancer patients are associated with cachexia, and IL-6 inhibitors have been suggested as possible treatment adjuncts in cancer patients in order to prevent this catabolic effect [27].

In line with the documented ability of TCZ to induce dyslipidemia, we observed significantly elevated levels of total cholesterol, HDL, and TG, as well as a rise in LDL that did not reach statistical significance in our RA patients following four months of TCZ treatment. While the elevated lipid profile raises concerns about its potential to increase CVD risk in RA patients, we did not detect changes in the two atherogenic indices we evaluated. Notably, atherogenic indices fluctuate less among RA patients and are considered more reliable in the assessment of CVD risk in these patients [28,29]. This finding is consistent with previous studies reporting elevation in the lipid profile with no change in atherogenic indices following TCZ treatment [23,28,30,31]. Moreover, the increase in HDL, which is considered to have a cardioprotective role in the general healthy population, could potentially mitigate this concern. The reported increase in RA patients of HDL particles, which might possess anti-inflammatory and athero-protective properties, indicates that the effects of TCZ on CVD risk are complex and still unclear [32].

Adipokines, and particularly leptin, are important mediators of the pathophysiology of RA and its comorbidities [10,12,33]. Xibillé-Friedmann et al. found that leptin, but not adiponectin, levels predict disease activity and response to conventional DMARD treatment in RA patients [34]. Indeed, many studies have shown elevated serum levels of adiponectin, leptin, and resistin in RA patients relative to healthy controls [3,4,6,7,9,10,11,12,13]. However, the literature is inconsistent regarding the effects of TCZ treatment on serum levels of adipokines in RA patients. We found that adiponectin levels decreased following therapy, while resistin levels and LAR increased following therapy. In contrast, Fioravanti et al. showed that after six months of TCZ treatment, resistin and leptin serum levels were not significantly changed, but the adiponectin level was increased [24]. Tournadre et al. showed no change in resistin and adiponectin levels, but a reduction in leptin serum levels [35]. Schultz et al. demonstrated that after three months of treatment, TCZ increased adiponectin levels, but did not change leptin levels, leading to a reduction in LAR [23]. In comparison, other treatments, such as infliximab (anti-TNFα), were found to decrease resistin levels [36], or did not have any effect on leptin and adiponectin levels in patients with RA [37,38]. Thus, the effects of TCZ are not straightforward, and may depend on additional, yet unknown factors.

Most of the above noted studies only compared the adipokine levels in RA patients before and after TCZ treatment. Therefore, it is noteworthy that we show here that in comparison with healthy controls, TCZ treatment normalized adipokine levels. The ability of TCZ to normalize adiponectin and resistin levels and to bring them closer to levels found in the healthy control group demonstrates a positive effect of TCZ on metabolism, in addition to its anti-inflammatory properties, and may hint at a putative cardioprotective role of this biologic agent. However, the increase in LAR and resistin levels, which have been shown to have pro-atherogenic and insulin resistant effects [5,14,17], cast doubt on this supposition.

Possible explanations for the differences between our study results and those of other studies include small sample sizes, demographic differences such as age and race, differences in disease duration, and treatment. BMI differences between study populations in the different studies may also be significant because BMI directly impacts leptin levels [6,10,23,34]. Of note, there were no significant changes in conventional DMARDs or prednisone dose in our RA patients throughout the study, suggesting that these medications were not confounding factors in our assessments.

Finally, we show that the change in the adipokine levels did not correlate with most of the known biochemical parameters that are associated with increased CVD risk or with RA disease activity. The only exception was the positive correlation found between the changes in HDL and leptin levels. HDL levels were previously found to negatively correlate with leptin and positively correlate with adiponectin in a healthy cohort, suggesting a link between leptin, insulin resistance, and the metabolic syndrome, as well as a cardioprotective role for adiponectin [39]. However, such a link would imply that other CVD risk factors, such as LDL, total cholesterol, or TG levels, should also correlate with leptin levels, which was not found in our study. Therefore, it is likely that the positive correlation between HDL and leptin levels that we observed is not representative, probably resulting from the small patient cohort. In contrast, the lack of correlation between adipokine levels with other parameters of CVD risk that we observed is similar to a previous study [24]. Taken together, our findings do not support a direct link between the adipokines leptin, resistin, and adiponectin and the reduction in CVD risk observed following initiation of TCZ treatment in RA patients, and may suggest that other adipokines or cytokines may play a greater role in mediating this effect, as was recently suggested [24], or that other mechanisms play a cardioprotective role in RA patients treated with this biologic agent.

Our results should be viewed with certain limitations, specifically the short follow-up period of four months and the relatively small sample size. Our study analyzed serum levels of only a few selected adipokines, and other adipokines could be included in a larger study. Because of the variation between serum and synovial fluid concentrations of various pro-inflammatory and anti-inflammatory mediators in RA patients [40], future studies should also include a comparison between serum and synovial fluid adipokine levels.

In conclusion, the impact of TCZ treatment on the metabolic profile of RA patients is complex. When taking all the changes recorded in our patients’ lipid and adipokine profiles into consideration, the following findings should be highlighted: the rise in HDL, the lack of worsening in atherogenic indices, and the normalization of adiponectin and resistin levels in TCZ responders in comparison with healthy controls. These changes suggest a protective role for TCZ on the metabolic and cardiovascular burden associated with RA, but still do not provide a mechanistic explanation for this phenomenon.

## 4. Methods

### 4.1. Study Population

Forty RA patients with active disease who were suitable candidates for TCZ treatment were recruited from rheumatology clinics in two medical centers in Israel. All patients met the 2010 American College of Rheumatology/European League Against Rheumatism (ACR/EULAR) RA criteria [41]. Patients were treated with 8 mg/kg TCZ infusions every four weeks. All other medical decisions were at the treating physicians’ discretion. Patients with diagnosis of another inflammatory arthritis or neoplastic disease were excluded. Parameters collected at baseline and after four months of TCZ treatment included demographic data, height, weight, calculated body mass index (BMI) (weight/height^2^), patient comorbidities with emphasis on CVD risk factors, current medications, and physical examination data with a focus on swollen and tender joint counts.

Laboratory variables studied included complete blood count and chemistry panel, lipid panel, CRP, and rheumatoid factor (RF), all analyzed by standard methods in a central laboratory. Total cholesterol, high density lipoprotein (HDL), and triglyceride (TG) levels were used to calculate the atherogenic index (AI, total cholesterol/HDL) and the AI of plasma (the base 10 logarithm of TG/HDL). Blood samples were obtained for the analysis of adipokines (leptin, adiponectin, and resistin), as well as for measurement of IL-6 levels and high sensitivity CRP (hsCRP). Leptin and adiponectin levels were used to calculate the LAR.

The control group of 40 volunteers without inflammatory diseases, renal failure, CVD or malignancies was age-and gender-matched with the RA group. Blood samples from this group were drawn at recruitment and tested for leptin, adiponectin, resistin, LAR, IL-6, and hsCRP levels.

The study protocol was approved by the local institutional review boards of the participating medical centers, and informed consent was obtained from all participants at recruitment (approval number at Carmel Medical Center CMC-0018-11 on 23 March 2011; approval number at Bnei Zion Medical Center BNZ043-11 on 27 July 2011).

### 4.2. Assessment of Clinical Response

Clinical response was assessed using the disease activity score-28 joint count (DAS28-CRP) and clinical disease activity index (CDAI) scores. Patients were categorized as responders by DAS28-CRP if their score was reduced by more than 1.2 points (∆DAS28-CRP ≥ 1.2), and their total DAS28-CRP score was <5.1 following four months of treatment; this represents moderate-to-large response to therapy according to the EULAR response criteria [42]. Patients were categorized as responders by CDAI if they demonstrated a reduction of at least one disease activity class after four months of treatment (i.e., from high disease activity (CDAI > 22) to moderate disease activity (CDAI 10–22) or from moderate disease activity to low disease activity or remission (CDAI < 10) [43,44].

### 4.3. Biochemical Analysis

Blood samples were drawn at baseline and after completing four months of TCZ therapy, immediately centrifuged at 1200 rpm, and stored at −80 °C until analysis. Serum concentrations of leptin, adiponectin, resistin, and IL-6 were determined using DuoSet enzyme-linked immunosorbent assay (ELISA) kits (R&D systems, Minneapolis, MN, USA), and hsCRP concentrations were determined using the hsCRP enzyme-linked immunosorbent assay (ELISA) kit (AssayPRO, St. Charles, MO, USA), according to manufacturer’s instructions.

### 4.4. Statistical Analysis

Continuous data are presented as mean ± standard deviation (SD) and median; categorical variables as numbers and percentages. The changes in the DAS28-CRP and CDAI scores, total cholesterol, HDL, TG, weight, and BMI levels between baseline and follow-up visits in RA patients were analyzed by paired t-test.

ANCOVA models adjusted to baseline BMI and statin treatment were used to compare the log-transformed adipokine concentrations in the RA and control groups. Mann–Whitney test was used to compare serum concentrations of IL-6 and hsCRP between the two groups, as appropriate. Correlation between the changes in adipokine levels and clinical and biochemical parameters of RA reflecting CVD risk before and after four months of TCZ treatment was calculated using Pearson’s correlation coefficient. The changes in adipokine serum concentrations in the RA group between baseline (before treatment) and follow-up time, four months after TCZ treatment, adjusted to BMI, statin treatment, and RA disease duration, were analyzed by linear mixed models with repeated measures. All tests were two-sided and *p* values of <0.05 were considered statistically significant. All statistical analyses were done using SPSS, version 24.0 (IBP Corp. Released 2016, IBM SPSS Statistics for Windows, version 24.0. Armonk, NY, USA: IBM Corp).

## Figures and Tables

**Table 1:** Patients demographic and clinical characteristics according to disease activity at baseline (disease activity score-28 joint count (DAS28)-C-reactive protein (CRP)).

Characteristic	Moderate Disease Activity (DAS ≤ 5.1) (*n* = 15)	High Disease Activity (DAS > 5.1) (*n* = 25)	Total (*n* = 40)	*p* Value
Age (years)	60 ± 14.96	56.04 ± 7.90	57.53 ± 11.07	NS
Gender (% female)	13 (86.7%)	20 (80%)	33 (82.5%)	NS
RF positive (%)	8 (53.3%)	13 (54.2%)	21 (53.8%)	NS
Anti-CCP positive (N) (%)	6 (10) (60.0%)	6 (13) (46.2%)	12/23 (52.2%)	NS
Disease duration (years)	6.46 ± 5.71	8.44 ± 5.52	7.7 ± 5.6	NS
**Concomitant Diseases**				
Ischemic heart disease (%)	1 (6.7%)	0 (0.0%)	1 (2.5%)	NS
Diabetes mellitus (%)	3 (20.0%)	5 (20.0%)	8 (20.0%)	NS
Hypertension (%)	5 (33.3%)	7 (28%)	12 (30%)	NS
Chronic obstructive lung disease (%)	1 (6.7%)	0 (0%)	1 (2.5%)	NS
Dyslipidemia (%)	6 (40.0%)	13 (52.0%)	19 (47.5%)	NS
Prior malignancy (%)	1 (6.7%)	0 (0.0%)	1 (2.5%)	NS
**Therapy at Baseline**				
Methotrexate	8 (53.3%)	19 (76.0%)	27 (67.5%)	NS
Sulfasalazine	1 (6.7%)	4 (16.0%)	5 (12.5%)	NS
Hydroxycholoroquine	1 (6.7%)	3 (12.0%)	4 (10.0%)	NS
Leflunomide	0 (0.0%)	3 (12.0%)	3 (7.5%)	NS
Corticosteroid dose in milligrams (mean + SD)	3 ± 5.92	7.2 ± 10.52	5.63 ± 9.22	NS
Statin use	6 (40%)	15 (56%)	20 (50%)	NS
Anti-diabetic therapy	3 (20.0%)	5 (20.0%)	8 (20.0%)	NS

* anti-CCP = anti-cyclic citrullinated peptide, (N) = number, NS = not significant, RF = rheumatoid factor, SD = standard deviation.

**Table 2:** Effect of tocilizumab (TCZ) on clinical and laboratory parameters.

Parameter	Before Treatment	After 4 Months of Treatment	*p* Value
**Inflammation Scores and Markers**			
DAS28-CRP score	5.45 ± 1.06	3.46 ± 1.37	<0.0001
CDAI score	36.59 ± 12.48	20.0 ± 12.56	<0.0001
28-tender joint count	12.48 ± 6.47	5.93 ± 5.21	<0.0001
28-swollen joint count	9.25 ± 5.39	4.06 ± 4.18	<0.0001
Patient global assessment of disease activity (0–100 mm)	76.45 ± 19.15	56.78 ± 23.18	<0.0001
Provider global assessment of disease activity (0–100 mm)	72.1 ± 15.5	44.1 ± 26.1	<0.0001
CRP (mg/dL)	1.33 ± 1.8	0.13 ± 0.42	<0.0001
hsCRP (mg/dL)	3.37 ± 2.0	0.74 ± 1.36	<0.001
IL-6 (pg/mL)	28.4 ± 94.42	69.28 ± 109.05	<0.001
**Anthropometric Measurements**			
Weight (kg)	74.5 ± 17.52	75.31 ± 16.91	NS*
BMI (kg/m^2^)	27.77 ± 6.6	27.96 ± 6.45	NS
**Lipid Profile and Atherogenic Indices**			
Total cholesterol (mg/dl)	199.4 ± 52.71	220.83 ± 53.39	0.003
LDL (mg/dl)	123.67 ± 36.87	131.38 ± 36.95	NS
HDL (mg/dl)	54.53 ± 19.0	59.0 ± 23.27	0.039
TG (mg/dl)	139.56 ± 68.53	167.67 ± 106.93	0.04
AI (HDL/cholesterol)	3.79 ± 0.78	3.96 ± 1.13	NS
AI of plasma [log(TG/HDL)]	0.93 ± 0.09	0.95 ± 0.11	NS
**RA Therapy**			
Prednisone (Mean ± SD)	5.63 ± 9.21	5.13 ± 8.28	NS
Methotrexate (%)	27 (67.5%)	25 (62.5%)	NS
Sulfasalazine (%)	5 (12.5%)	1 (2.5%)	NS
Hydroxychloroquine (%)	4 (10%)	3 (7.5%)	NS
Leflunomide (%)	3 (7.5%)	1 (2.5%)	NS

AI = atherogenic index, BMI = body mass index, CDAI = clinical disease activity index, CRP = C-reactive protein, hsCRP = high sensitivity CRP, DAS28-CRP = disease activity score-28 joint count, HDL = high density lipoproteins, IL-6 = interleukin-6, LDL = low density lipoproteins, SD = standard deviation, TG = triglycerides. * not significant.

**Table 3:** Serum adipokine levels in healthy controls compared with RA patients before and after four months of tocilizumab (TCZ) treatment.

Cytokine	Control	Patients before Treatment	Patients after Treatment	*p* Value		
				Control vs. Patient before treatment*	Control vs. Patients after treatment*	Patients before treatment vs Patents following four months of treatment**
Adiponectin (ng/mL)						
Mean (± SD)	3.75 ± 1.63	5.59 ± 2.39	4.53 ± 2.12	<0.0001	0.17	0.0001
Median	3.56	5.33	4.44			
Leptin (pg/mL)						
Mean (± SD)	21.92 ± 21.63	25.15 ± 26.36	29.36 ± 30.25	0.84	0.92	0.125
Median	15.05	19.38	15.95			
Resistin (pg/mL)						
Mean (± SD)	21.53 ± 8.19	16.25 ± 7.17	20.42 ± 8.06	0.63	0.63	0.001
Median	20.24	14.73	19.91			
Leptin/ Adiponectin ratio						
Mean (± SD)	6.44 ± 6.44	5.52 ± 6.08	7.99 ± 7.84	0.03	0.59	0.002
Median	4.36	3.93	4.90			

SD = standard deviation; * *p* value adjusted to BMI and statin; ** *p* value adjusted to BMI, statin treatment, and disease duration.

**Table 4:** Correlation between the changes in adipokine levels and clinical and biochemical parameters of RA reflecting cardiovascular disease (CVD) risk and disease activity, before and after four months of TCZ treatment.

		ΔDAS28	ΔCDAI	ΔhsCRP	ΔHDL	ΔLDL	ΔTG	ΔCholesterol	ΔAI	ΔAI-Plasma
**ΔAdiponectin**	*r*	0.026	0.020	−0.045	0.160	0.162	−0.005	0.257	0.106	−0.085
	*p*	0.875	0.902	0.782	0.365	0.393	0.979	0.135	0.549	0.633
**ΔLeptin**	*r*	0.186	0.025	−0.009	0.390	0.208	−0.008	0.200	−0.196	−0.217
	*p*	0.250	0.879	0.957	0.023	0.270	0.964	0.248	0.267	0.218
**ΔLAR**	*r*	−0.026	−0.078	−0.041	0.177	0.157	0.013	0.000	−0.194	−0.121
	*p*	0.876	0.631	0.803	0.318	0.408	0.938	1.000	0.273	0.494
**ΔResistin**	*r*	0.068	0.096	−0.058	0.009	0.240	−0.086	0.165	0.097	−0.008
	*p*	0.679	0.555	0.722	0.961	0.201	0.619	0.345	0.586	0.963

AI = atherogenic index, CDAI = clinical disease activity index, hsCRP = high sensitive CRP, DAS28-CRP = disease activity score-28 joint count, HDL = high density lipoproteins, LDL = low density lipoproteins, SD = standard deviation, TG = triglycerides. *r* = Pearson’s correlation coefficient, *p* = significance value.
